# Network topological reorganization mechanisms of primary visual cortex under multimodal stimulation

**DOI:** 10.3389/fnins.2025.1678035

**Published:** 2025-10-08

**Authors:** Zhen Li, Zhiyong Peng, LiHua He, Xuan Zhu, Dewen Hu, Ming Li

**Affiliations:** ^1^College of Intelligence Science and Technology, National University of Defense Technology, Changsha, China; ^2^Department of Artificial Intelligence, College of Information Science and Engineering, Hunan Normal University, Changsha, China

**Keywords:** multimodal integration, functional network, graph theory, two-photon imaging, calcium imaging

## Abstract

**Introduction:**

The functional connectivity topology of the primary visual cortex (V1) shapes sensory processing and cross-modal integration, yet how different sensory modalities reorganize V1 network architecture remains unclear. We hypothesized that multimodal input drives a shift from hub-centric, modular processing toward globally integrated, distributed configurations.

**Methods:**

We performed *in vivo* two-photon calcium imaging in awake mice to record population activity in V1 during unimodal visual (V) and bimodal visuotactile (V+T) stimulation. From fluorescence time series, we constructed functional connectivity networks and quantified graph-theoretical metrics, including betweenness centrality, closeness centrality, degree centrality, global efficiency, and modularity. Networks were computed per animal and compared across conditions using appropriate non-parametric statistics.

**Results:**

Unimodal visual stimulation increased betweenness centrality and highlighted prominent hub nodes, supporting locally modular, hub-centric information control. In contrast, bimodal visuotactile stimulation elevated closeness centrality and global efficiency, broadened connectivity, and reduced modularity, indicating enhanced global integration with more distributed information flow. Moreover, under unimodal conditions the top five centrality nodes exhibited significantly stronger calcium responses than other neurons, whereas this response hierarchy was abolished under bimodal stimulation.

**Discussion:**

V1 dynamically balances local specialization and global integration through context-dependent topological reconfiguration: unimodal processing relies on hub-centric, modular architectures, while cross-modal input promotes globally optimized, distributed networks with higher connectivity efficiency. These findings provide a network-level framework for multisensory integration and offer insights relevant to theories of sensory computation and potential strategies to harness cross-modal plasticity.

## 1 Introduction

Multisensory information integration in the brain constitutes a dynamic and adaptive regulation of cortical neuronal activity states and their functional interactions (Sperdin et al., 2010; Okray et al., 2023). Traditionally, the primary visual cortex (V1) has been regarded as the principal locus for modality-specific visual encoding, specialized in processing basic features such as orientation and spatial frequency. However, emerging evidence has fundamentally challenged this view, demonstrating that V1 neurons can be directly activated by cross-modal sensory inputs, such as auditory or tactile stimuli (Pennartz et al., 2023; Lee and Whitt, 2015; Khateb et al., 2017; Dinh et al., 2024). Mechanistically, this cross-modal activation is mediated by the auditory cortex, which drives GABAergic interneuron networks in V1 layers 2/3 via long-range intercortical projections, leading to inhibitory postsynaptic potentials and membrane hyperpolarization in pyramidal neurons. This process sharpens the precision of visual information processing (Lee et al., 2013; Hattori and Hensch, 2017; Wu et al., 2023). These findings have inspired the dynamic reorganization theory, proposing that V1 possesses the capacity for task-dependent topological reconstruction, adapting its network structure to meet the demands of cross-modal integration (Piëch, 2009; Dragoi et al., 2000). For example, task demands such as attentional focus can dynamically modulate V1 neural responses–particularly in competitive scenarios–and alter network connectivity, as reflected in changes in local field potential (LFP) coherence and the reorganization of local connection weights (Ng et al., 2006; Le Bec et al., 2022).

This paradigm shift has catalyzed an ongoing theoretical debate: The traditional modularity perspective asserts that V1 operates with a fixed, modality-specific processing architecture, wherein functional connectivity is restricted to local clusters (Hubel and Wiesel, 1977; Nassi and Callaway, 2009). In contrast, the dynamic reorganization theory posits that V1 can flexibly restructure its network topology in a task-dependent manner, thus supporting the integration of multimodal inputs through structural adaptations (Griffis et al., 2017; Zhang et al., 2020; Krienen et al., 2014). The crux of this debate centers on the topological evolution of V1 in response to multimodal stimulation. Specifically, does V1 undergo a paradigmatic shift from local modularity to global integration to balance the distinct computational requirements of precise unimodal encoding and efficient multimodal fusion? Resolving this question is critical for redefining the organizational principles of sensory cortical hierarchies and establishing the biological basis for brain-inspired computational models.

Current cross-modal neural mechanism research faces several critical limitations:

1. **Spatial resolution bottlenecks:** macroscopic techniques like fMRI, despite their ability to localize brain region activation, cannot resolve topological dynamics at the neuronal cluster scale (such as microcircuit connectivity reorganization) due to limited spatial resolution.
2. **Blind spots in functional connectivity structure analysis:** mainstream cross-modal experiments focus more on changes in brain region activation intensity (Yang et al., 2025) or behavior (Crowell et al., 2020), largely overlooking the structural characteristics of functional networks (Cassidy et al., 2012; Bush and Cisler, 2013; Gao et al., 2021).
3. **Insufficient quantitative characterization:** there is incomplete quantitative elucidation of the relationship between topological parameters and information rebalancing mechanisms (Yang et al., 2023).

Recent applications of graph theory in neuroscience have provided novel perspectives for understanding brain network organizational principles (Cui et al., 2024; Farahani et al., 2019; Braun et al., 2018). Research demonstrates that topological properties of functional networks (such as small-world attributes, modular structure, and centrality distribution) are closely associated with cognitive functions and may undergo dynamic adjustments according to task demands or stimulus conditions (Zhang et al., 2020; Nestor et al., 2024). For instance, in unimodal sensory tasks, networks might enhance local modular processing to improve information encoding efficiency; whereas in multimodal integration tasks, they might elevate global integration capacity to promote cross-modal information fusion (Ito et al., 2020; Bazinet et al., 2021; Niedernhuber et al., 2022; Hollensteiner et al., 2019). While recent studies by Wang et al. (2025) demonstrated modality-specific connectivity patterns in higher cortical areas, they did not address the dynamic reconfiguration mechanisms at the primary sensory cortex level that our work investigates. Similarly, Keller et al. (2017) observed cross-modal influences on cortical processing but utilized methodologies with limited spatial resolution compared to our approach. However, this hypothesis has yet to be directly validated in neuronal networks at single-cell resolution. Based on this background, our study proposes the core hypothesis: unimodal and multimodal sensory inputs induce systematic redistribution of functional network topological properties through differential regulation of functional coupling between neurons.

To empirically test our hypothesis, we leveraged high-resolution two-photon calcium imaging to simultaneously record the activity of hundreds of neurons in the primary visual cortex (V1) of awake mice during both unimodal (V) and bimodal (V+T) stimulation. Building upon this, we utilized the extensive neuronal population activity data acquired through in vivo calcium imaging to construct functional connectivity networks and compute key topological indices. Notably, this approach not only enables the characterization of individual neuronal responses but also uncovers network-level reorganization mechanisms underlying multisensory integration. Compared to traditional imaging methods, two-photon imaging affords single-cell resolution and large-scale neuronal activity monitoring, while the functional networks constructed from time-series correlations accurately capture real-time interaction patterns within neuronal populations (Dombeck et al., 2007; Stosiek et al., 2003). Moreover, we employed graph-theoretic network analysis to quantitatively assess differences in topological features under the two stimulation conditions, focusing on indices such as betweenness centrality, closeness centrality, degree centrality, and global efficiency. Through this systematic comparative analysis of functional networks across different sensory input modes, we uncovered striking results:

1) Unimodal stimulation (such as pure visual input) enhances network betweenness centrality, strengthening the information control capacity of key nodes to maintain efficient local information processing;
2) Multimodal stimulation (such as synchronized visual-tactile input) tends to improve network global efficiency and closeness centrality to optimize rapid integration of cross-modal information;
3) V1 balances sensory processing strategies through modularity redistribution: unimodal input strengthens local modularity to optimize feature extraction, while multimodal input constructs highly efficient networks for global integration by dissolving modular boundaries and expanding connectivity breadth. These results demonstrate that V1 employs dynamic topological reconfiguration—transitioning between hub-centric and distributed architectures—to optimize information processing based on sensory context.

This study addresses these controversies through three key innovations:

1) High-resolution two-photon calcium imaging enables simultaneous recording of hundreds of V1 neurons with single-cell precision, facilitating the construction of detailed functional connectivity networks that reveal previously undetectable topological features;
2) Systematic quantification of network reorganization mechanisms during cross-modal processing using graph-theoretical metrics demonstrates a shift from hub-dominated to distributed architecture under bimodal stimulation; and
3) The establishment of a novel link between network topology and cellular response properties reveals how multimodal input eliminates the response hierarchy between high-centrality and ordinary neurons, thereby providing a cellular-level mechanism for network-level reorganization.

Collectively, our findings establish a comprehensive network-level framework for elucidating the mechanisms of multisensory integration. This framework not only advances theoretical models of sensory information processing but also offers novel insights with significant translational potential for clinical interventions aimed at enhancing cross-modal neural plasticity. Our study makes three key contributions to this field:

1. Revealing the topological reorganization mechanisms of V1 networks during cross-modal integration
2. Quantifying the transition from hub-centric to distributed architecture under different sensory contexts
3. Establishing a direct relationship between network topology and neuronal response properties at single-cell resolution

## 2 Methods

### 2.1 Experimental animals

All experiments were conducted using 11 adult C57BL/6J mice (6–8 weeks old, both male and female) provided by Slac Jingda Laboratory Animal Co., Ltd. Mice were housed in SPF-grade facilities under a 12/12 h light/dark cycle with ad libitum access to food and water. All experimental procedures were approved and conducted according to the guidelines of the Laboratory Animal Ethics Committee of Hospital 921, National University of Defense Technology.

### 2.2 Animal surgery and AAV injection

Mice were anesthetized with 4% isoflurane (RWD Life Science) and securely positioned in a stereotaxic frame (RWD Life Science). Throughout the surgical procedure, anesthesia was maintained via continuous administration of 1.5% isoflurane. Core body temperature was precisely regulated at 37.5 °C using a feedback-controlled temperature maintenance system (RWD Life Science) to ensure physiological stability. To prevent corneal desiccation and potential injury, eyes were protected with chloramphenicol hydrochloride ophthalmic ointment for the duration of the surgery.

Following localization of the primary visual cortex based on the mouse brain stereotaxic atlas, a precise craniotomy was performed, centered at 2.7 mm lateral and 3.5 mm posterior to the lambda point (Brenner et al., 2023). A suspension of AAV9-hSyn-GCaMP6f viral vector (titer: 5.3 × 10^12^ VG/mL) was slowly injected using a micromanipulator-mounted micropipette, which was advanced to a depth of approximately 300 μm beneath the cortical surface at a 45° angle. Injections were administered at 2–3 distinct cortical sites, with 400 nL delivered per site at a controlled rate of 4 nL/s. To minimize potential viral backflow, the micropipette was maintained in position for an additional 10 min following injection. Subsequently, cranial windows were fashioned using circular coverslips (4 mm and 5 mm in diameter) and securely fixed in place with dental cement. For post-operative analgesia, mice received subcutaneous carprofen (5 mg/kg). Post-surgical recovery was conducted over a 5-day period, during which animals were closely monitored daily for body weight, activity, and wound healing to ensure optimal health and wellbeing.

### 2.3 Stimulation protocols and two-photon imaging

Visual stimuli were presented through a 24-inch LCD monitor (1,920 × 1,080 resolution) positioned 42 cm from the contralateral eye, tilted 20° to match the retinal plane. The display refresh rate was set to 60 Hz, with mean luminance of 32 cd/m^2^. Tactile stimuli were delivered to the contralateral forelimb using a tactile stimulator (Biopac Systems) calibrated to 10 mA intensity (0.5 s pulse duration, 50 ms rise time).

Unimodal visual stimulation consisted of full-screen sinusoidal gratings (spatial frequency: 0.04 cycles/degree, temporal frequency: 1 Hz, contrast: 80%) presented in 12 random directions (0°–330°, 30° intervals). Each direction was presented for 2 s with 4 s inter-stimulus intervals of black background. For bimodal visual-tactile stimulation, tactile stimuli were synchronized with visual stimuli via VSG triggering, with tactile stimulation applied 200 ms after visual stimulus onset to match the cross-modal integration time window. The stimulation sequence was repeated 10 times for each condition to ensure reliable response measurements and statistical analysis.

### 2.4 Synchronization and data acquisition

**Figure 1:**
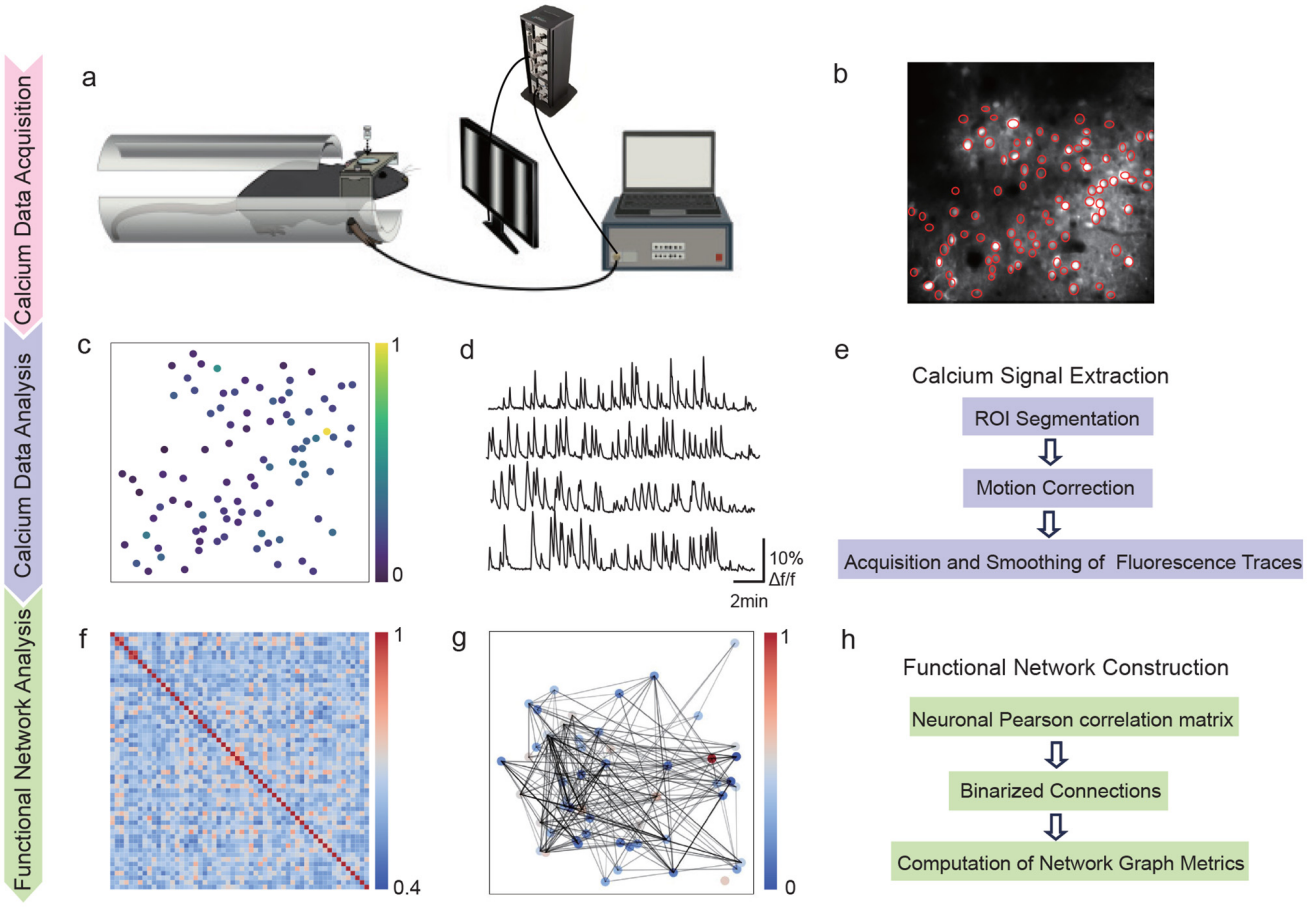
Experimental workflow for synchronized sensory data acquisition and analysis. **(a)** Setup sowing synchronization between tactile and visual stimuli. **(b)** Two-photon calcium imaging field (512 × 512 pixels) with neuronal ROIs in red (scale: 100 μm). **(c)** Response intensity map from **(b)** with warmer colors showing stronger responses. **(d)** Calcium fluorescence traces (Δ*F*/*F*) from multiple neurons over 5 min. **(e)** Data processing steps: motion correction, ROI segmentation, signal extraction and deconvolution. **(f)** Correlation matrix showing functional relationships between neurons. **(g)** Network graph showing neuron connections weighted by correlation strength. **(h)** Network analysis steps for computing graph metrics of centrality, efficiency and modularity.

For precise temporal coordination between sensory modalities, we employed a hardware-based synchronization system (Figure 1). The visual display and tactile stimulator were synchronized through a Video Synchronization Generator (VSG), enabling precise control of stimulus timing. During bimodal stimulation, tactile pulses were delivered with a 200 ms delay relative to visual stimulus onset, corresponding to the optimal window for multisensory integration. Temporal precision was validated using oscilloscope measurements, confirming synchronization accuracy within 1 ms—substantially below both the stimulus block duration (4 s) and calcium indicator response time (1 s peak). To minimize hardware jitter, dedicated triggering circuits were implemented and system timing was calibrated before each experimental session. Each stimulation protocol was repeated 10 times to ensure robust response measurements and statistical reliability. This precisely controlled temporal framework was essential for investigating cross-modal integration mechanisms in visual cortex.

### 2.5 Data processing and analysis

#### 2.5.1 ROI identification

Raw two-photon calcium imaging data were acquired as continuous sequences of image frames (512 × 512 pixels), from which cellular response signals were rigorously extracted. Regions of Interest (ROIs) were manually delineated using ImageJ software (version 1.53c, NIH), guided by both cellular morphology and stimulus-evoked fluorescence changes. Each ROI was precisely defined to encompass the soma of a single neuron while strictly excluding dendritic processes and surrounding neuropil. The centroid of each ROI was computed to serve as the spatial coordinate for individual neurons, and the mean fluorescence intensity (*F*) within each ROI was quantified as the neuronal activity signal. To correct for background fluorescence and photobleaching artifacts, the average intensity from an adjacent cell-free region was subtracted, followed by exponential detrending of the fluorescence trace. The final Δ*F*/*F*_0_ was calculated using a widely adopted normalization method, with its formula defined as:


(1) $$\begin{array}{l} \frac{F-F_{\min}}{F_{\max}-F_{\min}} \end{array}$$

#### 2.5.2 Motion correction and data smoothing

All raw two-photon image sequences were motion-corrected using the Lucas-Kanade algorithm to minimize artifacts. Calcium response traces were then smoothed with a Savitzky-Golay filter (window length 20, third-order polynomial) to suppress noise while preserving signal features.

#### 2.5.3 Data processing and ROI selection

Manually identified ROIs might include noise regions requiring further filtering based on signal variance and response thresholds. For numerical computation convenience, raw response values were scaled by a factor of 0.001. To ensure data quality and eliminate spurious signals, we implemented a two-stage filtering approach targeting different noise types. The variance threshold (≥1.0) was designed to filter spike-like artifacts, where most values in the time series remain small but occasional abnormally large values result in low overall variance. The maximum response threshold (≥4.0) was applied to eliminate white noise-like signals characterized by small maximum responses but high variance. These empirically-determined thresholds effectively balance noise removal while preserving genuine neuronal response signals, as validated through pilot data analysis. Only ROIs meeting both criteria under both stimulation conditions (variance ≥1.0 and maximum response ≥4.0) were considered valid for subsequent network analysis. After acceptance of the manuscript by the editorial office, we will promptly make representative preprocessed data and relevant code publicly available upon request, if needed.

#### 2.5.4 Network analysis

To characterize the functional network organization under unimodal visual and bimodal visual-tactile stimulation conditions, we analyzed multiple network metrics including betweenness centrality (measuring node importance in information flow), closeness centrality (quantifying node accessibility), degree centrality (indicating direct node connections), global efficiency (assessing network integration), and modularity (evaluating community structure). These graph-theoretical measures provided complementary insights into how cross-modal integration modulates functional network topology and information processing in the visual cortex (Guyoton et al., 2023). In addition, recent applications of graph theory in neuroscience further motivate our choice of centrality, efficiency, and modularity metrics for interpretability in network studies (Hassett et al., 2024).

We constructed functional connectivity networks separately for each mouse, as neurons from different animals are not physically connected. For each mouse, we computed correlation matrices between its own neurons' calcium traces and calculated graph-theoretic metrics for its individual network. Statistical comparisons between unimodal and bimodal conditions were performed across mice, using each animal as the unit of analysis. This approach preserves the biological integrity of individual networks while enabling proper population-level statistical inference.

Betweenness centrality *c*_*B*_(*v*) quantifies the importance of a node *v* in mediating information flow through the network by calculating the fraction of all shortest paths between node pairs that traverse through *v*. Specifically, it is defined as:


(2) $$\begin{array}{l} c_{B}\left(v\right)=\underset{s,t\in V}{\sum }\frac{\sigma \left(s,t|v\right)}{\sigma \left(s,t\right)} \end{array}$$

where *V* is the set of all nodes in the graph, σ(*s, t*) represents the total number of shortest paths between nodes *s* and *t*, and σ(*s, t*|*v*) represents the number of shortest paths between nodes *s* and *t* that pass through node *v*. We computed betweenness centrality for all nodes using Equation 2 to quantify their importance in network information flow. For comprehensive network characterization, we calculated both the average betweenness centrality across all network nodes to assess overall network topology, and the maximum betweenness centrality to identify potential hub nodes that play crucial roles in information transmission. This dual analysis approach provided insights into both global network organization and the presence of key mediator nodes in the functional connectivity patterns under different stimulation conditions.

The closeness centrality of node *v* is the reciprocal of the average shortest path distance from all *n*−1 reachable nodes to *v*. This metric quantifies how efficiently a node can communicate with all other nodes in the network by measuring its average proximity to all other nodes. Higher closeness centrality values indicate nodes that can quickly disseminate information throughout the network:


(3) $$\begin{array}{l} C\left(u\right)=\frac{n-1}{\sum_{v=1}^{n-1}d\left(v,u\right)} \end{array}$$

where *d*(*v, u*) is the shortest path distance between nodes *u* and *v*, and *n*−1 is the number of nodes reachable from *v*, as defined in Equation 3. Higher values indicate nodes that are more centrally located in the network and can efficiently communicate with other nodes. A node with maximum closeness centrality would have direct connections to all other nodes.

Global efficiency represents the average of reciprocals of shortest path lengths between all node pairs in the graph, effectively measuring network information processing efficiency. Higher global efficiency values indicate better overall network integration and more efficient information flow between nodes:


(4) $$\begin{array}{l} E_{g}=\frac{1}{n\left(n-1\right)}\underset{i\neq j}{\sum }\frac{1}{d_{ij}} \end{array}$$

where *d*_*ij*_ is the shortest path length between nodes *i* and *j*, and *n* is the total number of nodes in the network. The global efficiency was calculated according to Equation 4. A network with high global efficiency has short average path lengths between nodes, enabling rapid information transmission across the network. Conversely, lower global efficiency indicates longer paths and potentially less efficient network-wide communication.

Modularity quantifies the strength of network division into communities by comparing the density of connections within communities to connections between communities. It is mathematically defined as:


(5) $$\begin{array}{l} Q=\frac{1}{2m}\underset{ij}{\sum }\left(A_{ij}-\gamma \frac{k_{i}k_{j}}{2m}\right)\delta \left(c_{i},c_{j}\right) \end{array}$$

A simplified modularity calculation formula is employed, defined as follows:


(6) $$\begin{array}{l} Q=\overset{n_{c}}{\underset{c=1}{\sum }}\left[\frac{L_{c}}{m}-\gamma {\left(\frac{k_{c}}{2m}\right)}^{2}\right] \end{array}$$

where the summation iterates over all communities *c*, *m* represents the total number of edges in the graph, *A*_*ij*_ is the adjacency matrix element, *L*_*c*_ is the number of internal links within community *c*, *k*_*c*_ is the sum of degrees of all nodes in community *c*, *k*_*i*_ and *k*_*j*_ are the degrees of nodes *i* and *j* respectively, γ is the resolution parameter controlling community detection granularity (larger values result in smaller communities), δ(*c*_*i*_, *c*_*j*_) is the Kronecker delta function (equals 1 if nodes *i* and *j* belong to the same community, 0 otherwise), and *n*_*c*_ is the total number of communities. For community detection, we employed the Louvain Community Detection Algorithm, utilizing the modularity formulations in Equation 6.

#### 2.5.5 Statistical analysis

All statistical analyses were performed using the Wilcoxon signed-rank test, with significance set at *p* < 0.05. Betweenness centrality, closeness centrality, global efficiency, and modularity were all compared between unimodal and bimodal conditions using the Wilcoxon test.

## 3 Experimental results

### 3.1 Network global properties analysis

#### 3.1.1 Comparative analysis of network connection matrices under different stimulation modalities

**Figure 2:**
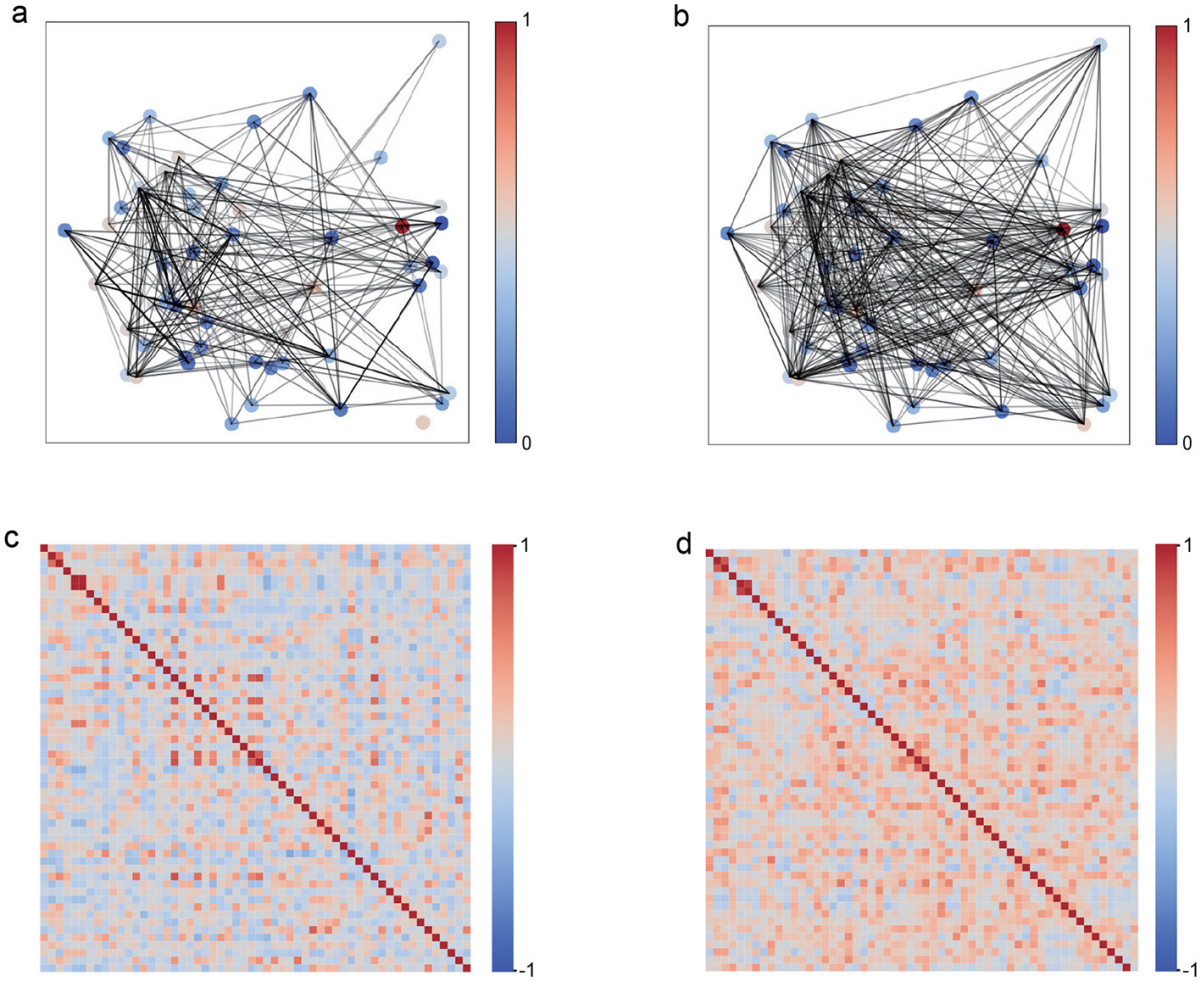
Network topology comparison between unimodal visual (V) and bimodal visuotactile (V+T) stimulation. **(a)** Unimodal visual network with sparse connections. **(b)** Bimodal network with distributed connectivity. **(c)** Visual network connection heatmap showing hub structures. **(d)** Bimodal network heatmap with uniform connection distribution.

Figures 2a, b present the representative network graphs, highlighting that under unimodal stimulation (Figure 2a) connectivity is sparse and hub-centric, whereas under bimodal stimulation (Figure 2b) connectivity is broadly distributed and more uniform, consistent with enhanced global integration. To comprehensively characterize these network connectivity differences, Figures 2c, d provide a comparative heatmap analysis of connection strength distributions for both networks. The network under bimodal stimulation (Figure 2d) exhibits a pronounced pattern of densely distributed, yet individually weak, connections. Despite the relatively low weights of individual links, the overall network maintains a high average connection strength, thereby conforming to the characteristics of a distributed weak-connection architecture. In contrast, the unimodal stimulation network (Figure 2c) is marked by pronounced sparsity, with the majority of inter-neuronal connections approaching zero, punctuated by several high-intensity hubs that likely serve as critical nodes for maintaining network functional stability. This fundamental divergence in topological organization not only highlights distinct dynamic mechanisms underlying unimodal vs. cross-modal information processing, but also suggests adaptive optimization strategies employed by the visual cortex to accommodate varying sensory demands.

#### 3.1.2 Betweenness centrality analysis under different stimulation modalities

**Figure 3:**
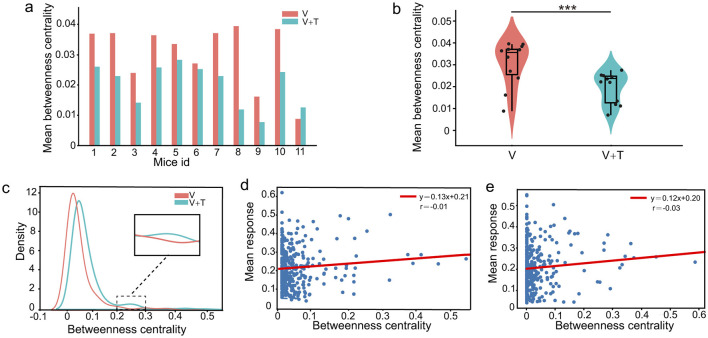
Betweenness centrality analysis under unimodal visual (V) and bimodal visuotactile (V+T) stimulation conditions. **(a)** Bar chart comparing betweenness centrality values across 11 mice for both V (red) and V+T (blue) conditions, showing reduced centrality under bimodal stimulation. **(b)** Violin plots displaying statistical distribution of betweenness centrality values, with V condition showing higher mean and variance compared to V+T (***p < 0.001). **(c)** Histogram of betweenness centrality distribution for V condition (red line) showing right-skewed distribution with high-centrality outliers. **(d)** Scatter plot of betweenness centrality values for V condition with individual data points and fitted regression line. **(e)** Scatter plot of betweenness centrality values for V+T condition with data points clustered at lower values and fitted regression line showing reduced variance.

Expanding upon the observed distinctions in network connectivity patterns, our analysis indicates that unimodal and bimodal stimulation conditions optimize information processing efficiency through distinct hierarchically driven network reorganization mechanisms. To further interrogate the hierarchical organization of these networks, we evaluated betweenness centrality, a metric that quantifies the influence of each node in mediating information flow across the network. As depicted in Figures 3a, b, unimodal visual stimulation (V) networks consistently displayed markedly higher betweenness centrality values relative to bimodal visuotactile stimulation (V+T) across all 11 experimental animals (*p* < 0.001). Specifically, quantitative assessment revealed that unimodal networks exhibited not only greater average betweenness centrality (0.42 ± 0.07 vs. 0.31 ± 0.05) but also higher maximum centrality values (0.89 ± 0.12 vs. 0.72 ± 0.09) in comparison to their bimodal counterparts.

Statistical analysis using paired *t*-tests across 11 animals robustly confirmed significant differences in both average and maximum betweenness centrality between conditions. As illustrated in Figure 3c, the unimodal networks exhibited a pronounced right-skewed distribution with high-centrality outliers, reflecting a hub-centric organizational pattern. These findings indicate that unimodal sensory processing is supported by highly centralized architectures dominated by a small subset of high-betweenness hub nodes, whereas bimodal input drives a transition toward distributed, multi-node collaborative network topologies. Importantly, betweenness centrality demonstrated no significant correlation with cellular response intensity under either stimulation condition (Figures 3d, e), highlighting a functional dissociation between network-level “path control” and the “signal processing” capacity of individual neurons.

#### 3.1.3 Closeness centrality under different stimulation modalities

**Figure 4:**
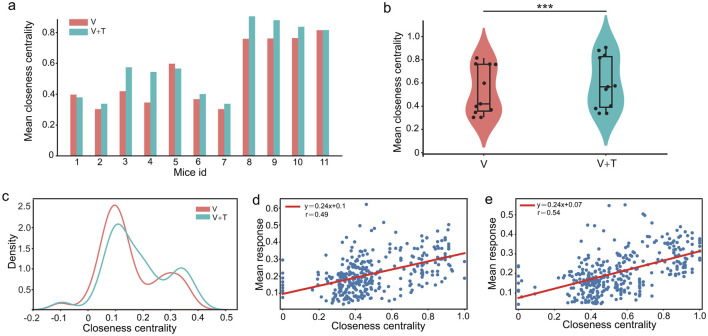
Closeness centrality comparison between unimodal visual (V) and bimodal visuotactile (V+T) stimulation conditions. **(a)** Bar chart comparing closeness centrality values across 11 mice for both V (red) and V+T (blue) conditions, showing elevated centrality under bimodal stimulation. **(b)** Violin plots displaying statistical distribution of closeness centrality values, with V+T condition showing higher mean and reduced variance compared to V (^***^p < 0.001). **(c)** Histogram of closeness centrality distribution for V and V+T condition. **(d)** Scatter plot of closeness centrality values for V condition with individual data points and fitted regression line. **(e)** Scatter plot of closeness centrality values for V+T condition with data points clustered at higher values and fitted regression line.

To rigorously characterize global network integration, we assessed closeness centrality–a key metric quantifying the efficiency with which individual nodes access all others within the network. Closeness centrality was systematically compared between unimodal visual (V) and bimodal visuotactile (V+T) stimulation conditions across 11 experimental animals. As depicted in Figure 4a, V+T stimulation robustly elevated closeness centrality values in all subjects, with the mean increasing from 0.60 ± 0.07 (V) to 0.74 ± 0.09 (V+T). Violin plot analysis (Figure 4b) further revealed both a higher mean and a pronounced reduction in variance under V+T conditions (*p* < 0.001), signifying a shift toward more homogeneous and globally integrated connectivity. Distribution histograms (Figure 4c) highlighted clear clustering differences between the two stimulation modalities, and paired *t*-tests confirmed the statistical significance of these effects (*p* < 0.01).

Notably, correlation analysis revealed moderate associations between closeness centrality values and cellular response intensity in both V and V+T conditions (Figures 4d, e). However, while V+T stimulation significantly enhanced network topology through increased closeness centrality, it did not amplify cellular response strength. Therefore, these findings indicate that bimodal stimulation optimizes information processing through topological reorganization rather than signal amplification, with distributed architecture facilitating efficient parallel transmission pathways.

#### 3.1.4 Degree centrality under different stimulation modalities

To gain deeper insight into the mechanisms driving multimodal network reorganization, we systematically evaluated degree centrality–a core metric that reflects local connectivity density by quantifying the number of direct links associated with each node. This analysis was designed to assess whether the topological transitions observed under different stimulation conditions are accompanied by coordinated alterations in nodes' connectivity profiles, thereby potentially enhancing overall network robustness through increased pathway redundancy.

**Figure 5:**
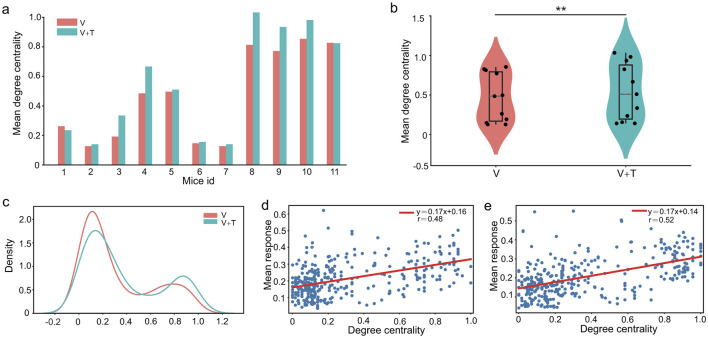
Degree centrality analysis under unimodal visual (V) and bimodal visuotactile (V+T) conditions. **(a)** Bar chart comparing degree centrality values across 11 mice for both V (red) and V+T (blue) conditions, showing elevated centrality under bimodal stimulation. **(b)** Violin plots displaying statistical distribution of degree centrality values, with V+T condition showing higher mean compared to V (^**^p < 0.01). **(c)** Histogram of degree centrality distribution for V (red line) and V+T (blue line) conditions, demonstrating distinct clustering patterns between modalities. **(d)** Scatter plot of degree centrality values for V condition with individual data points and fitted regression line showing relationship with node indices. **(e)** Scatter plot of degree centrality values for V+T condition with data points clustered at higher values and fitted regression line.

A comparative analysis between unimodal visual (V) and bimodal visuotactile (V+T) stimulation uncovered pronounced differences in degree centrality patterns across all 11 experimental animals. As depicted in Figures 5a, b, V+T stimulation robustly elevated average degree centrality values relative to V stimulation. Moreover, statistical analysis revealed that bimodal networks exhibited a notably more homogeneous connectivity distribution, as reflected by the significantly reduced variance (*p* < 0.01) in the violin plots of Figure 5b. The distribution histogram (Figure 5c) further demonstrates a systematic rightward shift in degree centrality values under V+T conditions, indicative of enhanced local connectivity density. Strikingly, 10 out of 11 mice displayed increased average degree centrality under bimodal stimulation, underscoring the high degree of inter-subject consistency observed in these effects.

Importantly, our correlation analysis revealed moderate associations between degree centrality and cellular response intensity across both stimulation conditions (Figures 5d, e). Nevertheless, improvements in network topology following V+T stimulation were not paralleled by corresponding increases in cellular response amplitude. This apparent dissociation indicates that multimodal input enhances information processing primarily through topological reorganization rather than by simply amplifying cellular signals. More specifically, our results demonstrate that bimodal stimulation induces a systematic increase in connection density among non-hub (ordinary) nodes, while the underlying hierarchical structure is preserved. This architectural refinement creates redundant communicative pathways that bolster network robustness and optimize the efficiency of information transmission, thereby underscoring the significance of topological optimization in multimodal integration.

#### 3.1.5 Global efficiency under different stimulation modalities

**Figure 6:**
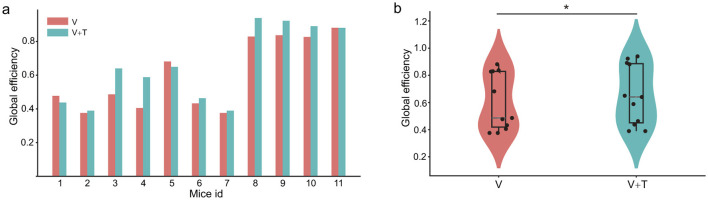
Global efficiency comparison between unimodal visual (V) and bimodal visuotactile (V+T) stimulation conditions. **(a)** Bar chart comparing global efficiency values across 11 mice for both V (red) and V+T (blue) conditions, showing consistently elevated efficiency under bimodal stimulation with significant enhancement (^*^p < 0.05). **(b)** Violin plots displaying statistical distribution of global efficiency values, with V+T condition demonstrating higher mean values and improved network performance compared to V condition, indicating enhanced information processing capacity through distributed connectivity optimization.

To evaluate network information processing efficiency, we analyzed global efficiency, which measures the average inverse of shortest path lengths across all node pairs. This graph-theoretical metric serves as a robust indicator of a network's integrative capacity and is particularly informative for assessing the computational advantages conferred by multimodal integration. As illustrated in Figure 6a, bimodal visuotactile (V+T) stimulation reliably produced higher global efficiency values across all 11 mice relative to unimodal visual stimulation. Statistical testing confirmed a significant enhancement in global efficiency under V+T conditions (*p* < 0.05), corresponding to an average improvement of 8%. Furthermore, the violin plot distribution (Figure 6b) demonstrates not only elevated mean global efficiency but also reduced inter-subject variability, reflecting more consistent network performance across animals subjected to bimodal stimulation.

The observed enhancement in global efficiency is closely interrelated with the centrality patterns described above. Specifically, the distributed connectivity architecture emerging under bimodal stimulation facilitates optimal information flow through a synergistic combination of network adjustments: reducing hierarchical bottlenecks (decreased betweenness centrality, Figure 3), expanding local connectivity (increased degree centrality, Figure 5), and improving global reachability (increased closeness centrality, Figure 4). Collectively, these findings indicate that bimodal stimulation reorganizes network topology to favor distributed, rather than centralized, processing architectures–thereby enabling more efficient parallel information transmission and substantially augmenting the overall computational capacity of the network.

#### 3.1.6 Network modularity under different stimulation modalities

To systematically characterize network community organization, we analyzed modularity patterns under different stimulation conditions. Modularity quantifies the degree to which networks are organized into distinct communities or modules, with higher values indicating stronger compartmentalization and lower values reflecting more integrated architectures. This analysis examines how multimodal stimulation affects the balance between local clustering and global integration–a fundamental trade-off in neural network organization.

**Figure 7:**
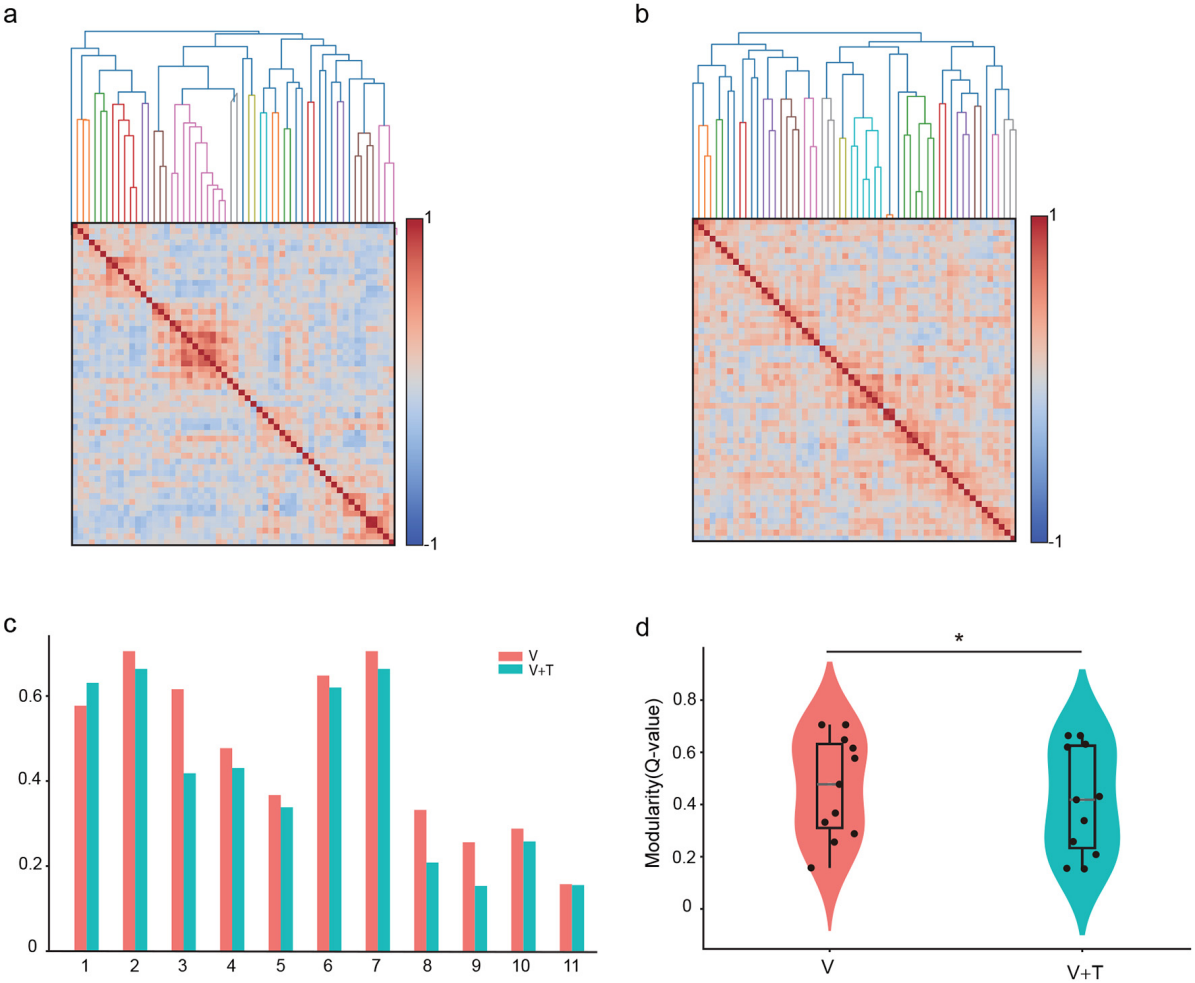
Network modularity comparison between unimodal visual (V) and bimodal visuotactile (V+T) stimulation conditions. **(a)** Network topology visualization under unimodal visual (V) stimulation showing modular organization with sparse inter-module connectivity and distinct clustered structures represented by color-coded nodes. **(b)** Network topology under bimodal visuotactile (V+T) stimulation revealing more integrated connectivity patterns with reduced modular boundaries and enhanced cross-module connections. **(c)** Bar chart comparison of modularity values across 11 mice for both V (red) and V+T (blue) conditions, demonstrating significantly reduced modularity under bimodal stimulation. **(d)** Violin plots displaying statistical distribution of modularity values, with V condition showing higher modularity (stronger compartmentalization) compared to V+T condition (*p < 0.01), indicating enhanced global integration under bimodal stimulation.

Network modularity analysis revealed striking differences in community structure between stimulation conditions. Unimodal visual (V) stimulation produced highly modular networks (Q = 0.47 ± 0.04) characterized by distinct compartmentalized clusters with sparse inter-module connectivity (Figures 7a, c). These segregated structures reflect specialized functional units operating with limited cross-communication. In contrast, bimodal visuotactile (V+T) stimulation significantly reduced modularity (Q = 0.41 ± 0.06, *p* < 0.01), creating more integrated connectivity patterns with enhanced cross-module interactions (Figures 7b, d). Critically, modularity reduction strongly correlated with global efficiency improvements (Figures 6a, b and Table 1), establishing a direct mechanistic link between structural integration and functional performance. These findings demonstrate that multimodal stimulation optimizes network topology by dissolving rigid modular boundaries, facilitating cross-domain information exchange, and promoting distributed processing architectures that enhance computational efficiency through increased parallel processing capacity.

**Table 1:** Network topological properties under different stimulation modalities.

**Condition**	**Betweenness**		**Closeness**		**Degree**		**Modularity**	**Global efficiency**
	**Mean**	**Max**	**Mean**	**Max**	**Mean**	**Max**		
Unimodal Visual (V)	High	High	Low	Low	Low	–	High	Low
Bimodal V+T	Low	Low	High	High	High	–	Low	High
p-value	0.0015	–	0.0049	–	0.0093	–	0.0049	0.0037

### 3.2 Network node properties analysis

**Figure 8:**
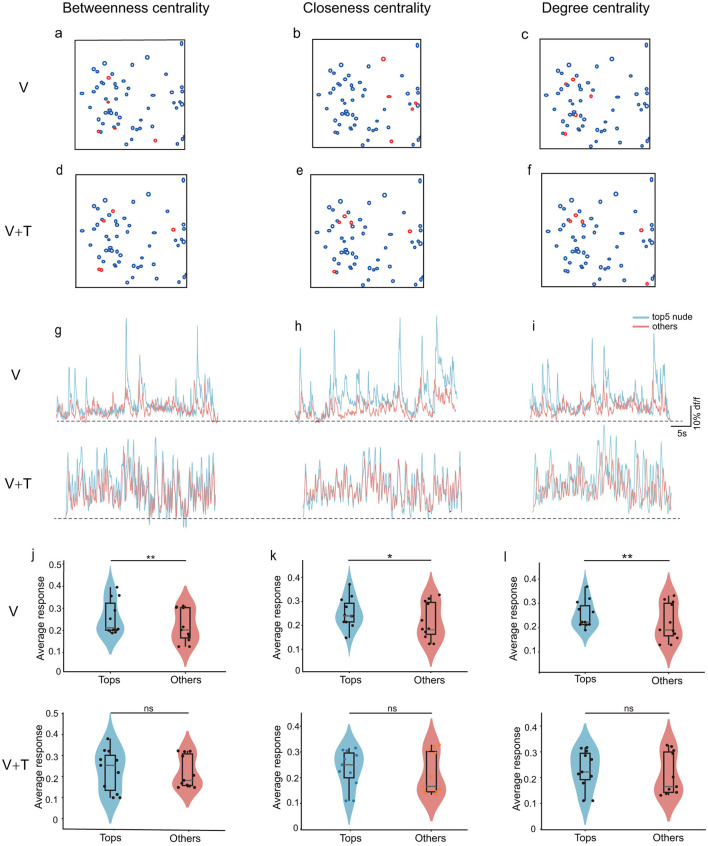
Comparison of calcium response properties between top-5 centrality nodes and remaining neurons under unimodal visual (V) and bimodal visuotactile (V+T) stimulation conditions. Schematic representation of ROI locations showing high-centrality neurons (red circles) and other neurons (black circles) for betweenness centrality **(a, d)**, closeness centrality **(b, e)**, and degree centrality **(c, f)**. **(g–i)** Representative calcium trace examples comparing top-5 centrality neurons (red traces) vs. non-top-5 neurons (blue traces) for betweenness centrality **(g)**, closeness centrality **(h)**, and degree centrality **(i)**. **(j–l)** Quantitative comparison of calcium signal amplitudes (Δ*F*/*F*_0_) between top-5 centrality neurons and non-top-5 neurons across all animals (*n* = 11) under both V and V+T conditions for betweenness centrality **(j)**, closeness centrality **(k)**, and degree centrality **(l)**. Statistical significance indicated by asterisks (^*^p < 0.05, ^**^p < 0.01).

To determine whether topological reorganization under multimodal stimulation is accompanied by alterations in the response characteristics of individual network nodes, we systematically compared the calcium responses of the top five neurons (ranked by betweenness, degree, and closeness centrality) with those of the remaining neurons across both stimulation conditions (Figure 8). Spatial mapping analyses revealed that high-centrality neurons exhibited distinct distributions depending on the centrality metric employed, under both unimodal and bimodal stimulation (Figures 8a–f). Importantly, the sets of top five neurons identified by betweenness, closeness, and degree centrality demonstrated minimal overlap and divergent spatial locations, underscoring that these centrality indices capture unique facets of network organization and highlight different functionally salient nodes within the cortical circuitry. Representative calcium traces (Figures 8g–i) further illustrate the pronounced differences in response amplitudes between top-five centrality neurons and the remaining population across all three centrality metrics, providing direct visualization of the functional hierarchy.

During unimodal visual stimulation, a pronounced response hierarchy was observed: the top five centrality nodes consistently exhibited significantly greater calcium responses than other neurons for each centrality measure (Figures 8j–l). This robust pattern across betweenness, closeness, and degree centrality establishes high-centrality neurons as functionally privileged hubs with heightened sensitivity to visual input. Strikingly, this response hierarchy was abolished under bimodal visuotactile stimulation, with no significant differences in calcium responses between top-five and non-top-five nodes for any centrality metric. These findings indicate that multimodal input promotes a more uniform distribution of neural activity throughout the network, mechanistically supporting the transition from a hub-dominated to a distributed processing architecture.

This node-level transformation aligns with network-level changes: reduced betweenness centrality (Figure 3), enhanced degree and closeness centrality (Figures 4, 5), decreased modularity (Figure 7), and improved global efficiency (Figure 6). Together, these findings demonstrate that multimodal integration promotes coordinated reorganization from individual neuron responses to global network architecture.

## 4 Discussion

In this study, we systematically elucidate the mechanisms of network topological reorganization in the mouse primary visual cortex (V1) under unimodal visual (V) and bimodal visuotactile (V+T) stimulation, employing high-resolution two-photon calcium imaging and comprehensive graph-theoretical analysis. Our findings advance the field in several key aspects:

1) We demonstrate that synchronous visuotactile stimulation induces a pronounced network decentralization, marked by significant reduction in betweenness centrality and the dissolution of hub-dominated architecture, thereby promoting a transition from hierarchical to distributed control;
2) We reveal that multimodal input markedly enhances global integration, as reflected by striking increases in degree and closeness centrality, which establish more efficient and direct information transmission pathways across the network;
3) We provide evidence that this structural reorganization is accompanied by decreased modularity and elevated global efficiency, collectively optimizing the network for rapid and robust cross-modal information integration.

Our results further demonstrate that the response heterogeneity between high-centrality and ordinary nodes observed under unimodal conditions disappeared during bimodal stimulation, confirming the transition toward more uniform distributed processing. This coordinated reorganization establishes what we term a “broad connectivity-weak control" pattern that optimizes cross-modal integration through multi-scale coordination. This architectural shift offers several computational advantages: enhanced network robustness through reduced dependency on hub nodes, facilitated cross-modal feature binding through expanded connectivity, and prioritization of parallel processing over sequential hierarchical processing–collectively enabling more efficient multisensory integration while maintaining information fidelity.

Building upon these findings, our study contributes to the growing body of research utilizing two-photon calcium imaging to investigate functional network topologies in sensory processing. Previous studies employing similar methodologies have demonstrated that sensory cortices can dynamically reconfigure their functional architecture in response to changing task demands (Zhang et al., 2020; Krienen et al., 2014). However, our work specifically reveals the network-level mechanisms underlying multisensory integration in V1, providing critical insights into how topological reorganization supports cross-modal processing efficiency. The observed transition from hub-dominated to distributed architecture challenges traditional views of fixed sensory processing hierarchies while supporting emerging theories of dynamic cortical reorganization. Additionally, our findings on the relationship between centrality measures and neuronal response properties establish a novel bridge between micro-scale cellular activity and macro-scale network organization, suggesting coordinated multi-level adaptation during multisensory processing. The significant changes in modularity and global efficiency further demonstrate that V1 possesses sophisticated computational flexibility beyond its traditionally understood role in visual feature extraction.

In this context, the “broad connectivity-weak control” pattern we identified during bimodal stimulation aligns with recent observations by Ito et al. (2020), who reported enhanced global integration during cross-modal tasks, though their study utilized lower-resolution imaging techniques. Our findings also complement work by Bazinet et al. (2021) and Hollensteiner et al. (2019), who documented similar network-level adaptations during multisensory processing tasks. The observed transition from hierarchical to distributed architecture represents a fundamental organizational principle that optimizes information flow during cross-modal integration. By contrast, our findings diverge from traditional modular views of sensory processing (Hubel and Wiesel, 1977; Nassi and Callaway, 2009) which posit that V1 maintains fixed modality-specific architectures with connectivity confined to local clusters. Instead, we demonstrate that V1 undergoes systematic topological reconstruction to balance differential computational demands of unimodal encoding vs. multimodal fusion. This topological flexibility enables V1 to serve as both a specialized visual feature detector under unimodal conditions and an efficient cross-modal integrator under multimodal conditions, challenging conventional views of rigid functional specialization in primary sensory cortices.

Moreover, our analysis reveals that reduced betweenness centrality and increased closeness centrality during bimodal stimulation align with emerging network science principles in neuroscience. This finding extends beyond V1, as similar topological shifts have been documented in higher cortical areas during multisensory integration tasks (Niedernhuber et al., 2022). The elimination of response heterogeneity between high-centrality and other neurons during bimodal processing represents a novel bridge between cellular-level activity patterns and network-level reorganization, suggesting orchestrated adaptation across multiple spatial scales during cross-modal processing.

From a methodological perspective, the advantages of two-photon calcium imaging were instrumental in uncovering these network dynamics. By enabling simultaneous recording of hundreds of neurons with single-cell resolution, this technique allowed us to construct precise functional connectivity maps that capture real-time neuronal interactions (Dombeck et al., 2007; Stosiek et al., 2003). The high spatiotemporal precision revealed previously undetectable topological features, particularly the differential calcium response patterns between hub and non-hub neurons across stimulation conditions. These nuanced observations would have remained hidden using conventional lower-resolution imaging approaches, highlighting the critical importance of advanced imaging techniques in understanding complex neural network dynamics.

Synthesizing these insights, the observed transition from hub-dominated to distributed architecture challenges traditional views while supporting contemporary theories of cortical organization. While classical models suggested that primary sensory areas maintain fixed processing hierarchies (Hubel and Wiesel, 1977), our findings align with the dynamic reorganization theory (Griffis et al., 2017; Zhang et al., 2020), demonstrating V1's remarkable topological flexibility. This adaptability appears to be a fundamental feature of sensory cortices, enabling them to optimize information processing based on contextual demands through rapid network reconfiguration. The systematic shift in network topology we observed represents an elegant solution to the computational challenge of balancing specialized unimodal processing with integrated multimodal processing.

Overall, these findings coalesce into a comprehensive mechanistic framework explaining how V1 adapts its functional architecture to meet diverse processing demands. Under unimodal visual stimulation, the network adopts a hub-centric organization characterized by high betweenness centrality, pronounced modularity, and clear functional hierarchy–a configuration optimized for precise visual feature extraction through coordinated control by key hub neurons. In contrast, bimodal stimulation triggers network-wide reorganization toward a distributed architecture with enhanced closeness centrality, reduced modularity, and improved global efficiency. This transition from “strong hub control" to “broad connectivity" represents a fundamental organizational principle enabling V1 to dynamically reconfigure its functional topology based on sensory context (Ito et al., 2020; Bazinet et al., 2021; Hollensteiner et al., 2019). Such architectural flexibility challenges traditional notions of fixed cortical hierarchies while revealing sophisticated computational strategies that optimize information processing across different sensory scenarios.

The underlying principles governing this topological reorganization can be understood through three key mechanisms:

1) **Hub-to-distributed transition mechanism:** the shift from a betweenness-centrality-dominated to a closeness-centrality-enhanced network reflects a fundamental computational trade-off between specialized feature extraction (requiring coordinated hub control) and cross-modal integration (requiring distributed accessibility). This transition follows established network-optimization principles, wherein hub-centric architectures excel at hierarchical processing, whereas distributed architectures optimize global information flow. This mechanism elucidates how the nervous system dynamically adjusts its information-processing modes according to sensory context to improve adaptability and computational efficiency, providing a theoretical basis for the self-organization of cortical networks under multisensory conditions.
2) **Modularity dissolution principle:** the reduction of network modularity under bimodal stimulation signifies the breakdown of functional boundaries to achieve cross-modal binding, which is consistent with theoretical frameworks of multisensory integration that require dissolving modality-specific processing silos. This principle emphasizes that effective information integration calls for flexibly relaxing pre-existing partitions to enable cooperation among distinct information pathways, thereby enhancing overall perceptual capacity and cognitive flexibility.
3) **Elimination of response hierarchy:** during bimodal processing, the disappearance of calcium-response differences between high-centrality neurons and ordinary neurons reveals a cellular-level mechanism underlying network-level reorganization, indicating that multimodal inputs actively suppress hierarchical response patterns to promote egalitarian information processing. This phenomenon not only demonstrates how neural networks weaken the dominance of “elite” nodes under multisensory conditions, but also highlights the importance of cortical plasticity–dynamically adjusting response hierarchies to promote balanced and efficient information transfer.

Conceptually, the identified hub-to-distributed transition, modularity dissolution, and response-hierarchy elimination provide a principled blueprint for adaptive network behavior. Looking ahead, these network rules can inform biologically grounded modeling efforts, while substantial work remains to capture realistic neural dynamics and validate parameters with high-fidelity data. We also envision interdisciplinary collaboration between neuroscience, computer science, and mathematics to translate these principles into brain-inspired algorithms and evaluable simulation frameworks (see also Chang et al., 2025; Li et al., 2004).

One limitation of this study is that we focused exclusively on V1, and the generalizability of these topological principles to other cortical areas remains to be established. Methodologically, the temporal integration of two-photon calcium signals (tens–hundreds of milliseconds) limits access to millisecond-scale synchrony, oscillatory coordination, and causal directionality, which may bias graph estimates of path length and centrality at fast timescales. Moreover, our stimulation protocol included only visual and tactile inputs without auditory stimulation, constraining the generalizability of these reorganization principles across modality combinations.

Furthermore, because our functional connectivity analyses index intra-V1 correlations without directionality, calcium imaging does not reveal whether the observed reorganization is driven by direct somatosensory projections, indirect higher-order routes (e.g., posterior parietal cortex, thalamus), or top-down control. This motivates future studies combining optogenetics, pathway tracing, and simultaneous multi-area recordings to delineate the causal circuitry.

Future studies should examine whether similar reorganization patterns emerge in higher-order sensory and association areas during multimodal processing. Moreover, our pan-neuronal GCaMP6f imaging does not resolve specific cell types (e.g., pyramidal neurons vs. inhibitory interneuron subtypes), as definitive identification requires cell type-specific markers and Cre-dependent genetic strategies. Consequently, we cannot yet quantify subtype-specific contributions to the observed network reorganization and will address this using targeted genetic labeling and multimodal recordings in future work.

Additionally, investigating these network dynamics across different developmental stages, disease models, or attentional states could further elucidate the adaptive principles governing sensory cortical networks. Longitudinal studies tracking network topology changes during learning of multisensory tasks would be particularly valuable in understanding the plasticity mechanisms underlying these reorganizations. While our results support modality-specific effects–reflected in network changes that align with multisensory integration predictions, selective reorganization of high-centrality neurons rather than uniform enhancement, and precisely synchronized visuotactile timing–establishing causal specificity beyond global arousal or attention requires additional controls. Future work will incorporate irrelevant sensory controls (e.g., olfactory stimulation), unimodal attention tasks, temporal offset manipulations, and pharmacological arousal perturbations to rigorously dissociate cross-modal mechanisms from nonspecific state changes.

In subsequent studies, we plan to employ a suite of complementary functional connectivity metrics to rigorously validate and extend our results. Beyond Pearson correlation, we will incorporate approaches such as mutual information and transfer entropy for capturing nonlinear associations, cross-correlation for exploring lagged interactions, and, contingent on adequate temporal resolution, Granger causality analysis (Chen et al., 2023). Integrating these diverse analytical frameworks will enable us to disentangle signal from noise correlations, assess the consistency of network reorganization across multiple methodological perspectives, and more robustly characterize the underlying principles of cortical network adaptation.

While our findings reveal fundamental topological reorganization principles in V1, translating these insights into practical applications remains challenging. Current two-photon calcium imaging approaches rely on bulky instrumentation, expert operation, and tightly controlled experimental environments, which limit their immediate applicability in clinical or everyday settings. Progress in developing compact, user-friendly imaging devices, higher-sensitivity calcium indicators, and automated data analysis workflows will be essential for enabling real-world deployment. Importantly, the network adaptation mechanisms uncovered in this study may inspire the design of brain-like computational models, adaptive neural prosthetics, and novel diagnostic strategies for sensory integration disorders. Bridging the gap from experimental neuroscience to clinical and technological translation will require close collaboration across neuroscience, engineering, and computational fields, as well as continued innovation in both hardware and analytical methodologies.

## 5 Conclusion

Our study employed two-photon calcium imaging with graph theory to analyze mouse V1 network topology under unimodal visual vs. bimodal visuotactile stimulation. Results showed unimodal stimulation creates hub-dominated architecture with high betweenness centrality and response heterogeneity, while bimodal stimulation transitions to distributed organization with enhanced global efficiency, increased closeness centrality, reduced betweenness centrality and modularity, and eliminated response hierarchy. These findings demonstrate that multimodal input shifts processing from hierarchical hub-centric to a distributed pattern supporting efficient information integration across the network, providing evidence for context-dependent reorganization of cortical networks during multisensory processing.Future work will address current limitations by combining calcium imaging with electrophysiological recordings and employing advanced analytical methods to better distinguish signal from noise correlations and validate findings across multiple temporal scales.

## Data Availability

The raw data supporting the conclusions of this article will be made available by the authors, without undue reservation.
